# Cinematographic Habits of Future Spanish Teachers from a Socio-Educational Perspective

**DOI:** 10.3390/ijerph17155361

**Published:** 2020-07-25

**Authors:** Alejandro Lorenzo-Lledó, Asunción Lledó, Elena Pérez-Vázquez, Gonzalo Lorenzo

**Affiliations:** Department of Development Psychology and Teaching, Faculty of Education, University of Alicante, 03690 San Vicente del Raspeig, Spain; asuncion.lledo@ua.es (A.L.); elena.pv@ua.es (E.P.-V.); glledo@ua.es (G.L.)

**Keywords:** cinema, film, cinematographic habits, mass media, education, future teachers, university students

## Abstract

One of the key features of today′s society is the role of technology and mass-media. Among these tools, cinema has influenced successive generations more than 100 years. From an educational point of view, it is a resource of high pedagogical value. Moreover, it is present in the daily life of university students who will be the future teachers. Therefore, the aim of this study is to find out the film consumption habits of students in the teacher′s degree in Spanish universities. A quantitative approach was adopted with a survey design, and the national sample was made up of 4659 students. They ware from the different regions of Spain and 58 universities. The questionnaire called Percepciones sobre las potencialidades del cine como recurso didáctico en las aulas de Infantil y Primaria (PECID) was designed ad hoc for this research. The results obtained showed that most students have a weekly habit of consuming films, with fiction being the most popular type of film. Likewise, the viewing of films is preferably done through television and the computer. It can be concluded that this familiarity with cinema outside the university should be complemented with specific training in the teacher′s degree. In this sense, it contributes to an optimal application of cinema.

## 1. Introduction

One of the essential features of today′s society is the presence of technology and audio-visual media, which influence the ways of interacting and perceiving reality. Ref. [1] indicated that this global phenomenon has shaped a cultural reality centered on the development of the image, as compared to a subtle abandonment of reading and writing. This context makes demands on citizens, given that the existence of a diversity of languages implies the need to dominate them in order to have full growth. Ref. [2] argued that literacy in the 21st century is a set of cultural practices that enables engagement and expression of meaning through mastery of different domains, such as speech, writing, still and moving images, and music.

Among the most relevant audio-visual media is the cinema, with more than 100 years of history. The cinema comprises a form of self-expression and has influenced many generations. Several works Ref. [3,4,5] have conceptualized cinema from different perspectives, such as art, technique, spectacle, language, document, entertainment, pictorial expression, source of information and culture. Ref. [6] highlights a double facet in the cinema. On the one hand, the cinema as an art form. On the other hand, as a mass media, as it reflects customs, fashions and beliefs. For their part, Ref. [7] pointed out that cinema is an art of complex expression as it is at once audio-visual, digital, multimedia, plastic, dynamic, verbal and gestural. All this increases its capacity to generate changes in the subjects. In this sense, cinema is a language with a great power of attraction that can provoke a process of identification in the spectator and stimulate him to perform behaviors for life and also for death [8].

In the history of cinema, a diversity of tendencies and topics have been shown, forming different themes and rules, which are recognized by the spectators. Therefore, although there is no agreed taxonomy around the different genres or types of cinema, we can highlight, in the first place, the fiction genres, which have in common stories and fictional characters that, as [9] indicates, base their verisimilitude on the internal coherence and narrative of the created world. In contrast, documentary cinema aims to capture fragments of reality in a faithful way and to connect with the being of the spectator and not with the wanting to be [10]. Similarly, it is important to mention animation cinema, which with the rapid technological advances and starting from an image of non-real action, has had a great development, offering entertainment, fun and a creativity that manages to seduce the spectator [11]. In animated film, it is worth noting the enormous influence exerted by Disney, as a pillar of entertainment and generator of cultural representations for children in many countries [12].

Considering the nature of cinema and its evolution throughout history, the language of film is eclectic and comes from many sources. Ref. [13] indicate that cinema is a compendium of communication elements. Ref. [14] states that cinema is the synthesis of many arts and disciplines. Nevertheless, cinema has its own identity, despite the interrelations it has with other languages and the fact that it forms part of a wider audiovisual ecosystem, and should not be confused with other media. In this sense, Ref. [2] considers cinema as a form of artistic expression, as opposed to other media, such as television, which are essentially entertainment, and advocate that cinema should not be diluted in the generic category of social media. Similarly, Ref. [15] claims the purity of cinema and denounces the contamination suffered by the audiovisual media, which ends up denaturing the essence of cinema. In this way, they indicate that “the word audiovisual should be removed from everything that specifically relates to cinema and advocate a radical separation” [15] (p. 56). In this line, the cinema, catalogued as the seventh art, is pure, and does not need to be critically analyzed with respect to other media, such as television or advertising. The present study promotes the identity of cinema itself, but without forgetting the growing connections between the various audiovisual languages. To this, it must be added the technological changes in film production and dissemination. If this is not taken into account, we would have a partial vision of cinema, eliminating elements of reflection on it. From this perspective, it is understood that a film is an audiovisual work conceived as a cinematographic production, whose main objective is to be shown in cinemas, but which can also be shown in other dissemination channels, such as television or the Internet, but which is different from other non-cinematographic audiovisual content.

From an educational point of view, if one of the objectives that every educational system should have is to generate in the students some competences for the free and autonomous discovery of the world in which they live, in favor of an integral formation, the cinema cannot be absent in the educational centers. Therefore, the cinema as a mirror of reality has a great pedagogical potential, since it is an attractive medium that, as [16] note, offers the opportunity to learn in a more exciting way and to reflect and exchange ideas on psychosocial realities. Ref. [17] emphasize that it is necessary to redefine cinema as an exceptionally enriching medium for educating in the image. To give it its true entity, cinema should not be understood as a simple didactic support, but as the shaper of new, more significant learning environments, since, following [18], cinema is a tool that, apart from exposing a visual representation, is loaded with other symbols, and that, if conducted in an optimal way, can generate a more enriching and collaborative language between the teacher and the student.

Among all the audiovisual media, the cinema, as well as the television, is the one that has a greater tradition of didactic use since their generalization in the middle of the 20th century [19]. For [20], the inclusion of cinema in education is justified because it can generate a durable learning, attract students and be interdisciplinary. Nonetheless, the specific application of cinema in education is not without its difficulties, which put the development of all its educational possibilities at risk. According to [21], the use of the cinema in education must be done in a very concrete way, with a correct integration in the curricular programming and linking it closely to the didactic objectives. This author concludes that it is essential to carry out an adequate planning and to delimit what is intended to be achieved through the cinema. Ref. [22] emphasizes that one of the most relevant aspects is knowing how to correctly choose the film to be worked on, carefully applying some guidelines. On the other hand, the inclusion of cinema is a task for school organization to determine the different roles, as well as for management, implementing the necessary strategies [23]. Ref. [24] indicates that the greatest challenges when applying cinema in the classroom are the lack of teacher training, the lack of knowledge on the part of the students, the lack of resources and support materials in the schools and the rigidity of the curriculum. In order to overcome time and resource constraints, Ref. [6,19] argue that it is desirable to take advantage of the flexibility offered by digital cinema and adapt films to the time available and educational objectives. In this sense, it is advisable to use whole movies, fragments of movies or short films and to stop the film, move it forward or backward depending on the activity being carried out [25]. Therefore, it can be said that the optimal application of cinema in the classroom essentially requires a pedagogical strategy for educational use, and the involvement of teachers and educational institutions.

In contrast to other resources with educational potential, cinema as a mass media is present in the lives of teachers, whether they are already in service or in their pre-service teacher education. If we look at the second group, it is worth asking what relation these students have to the cinema. The role that cinema has in its leisure and cultural activities is a socio-educational reality that deserves to be investigated as a variable to be taken into account in order to face the application of cinema in the classroom. In this sense, cinema as a social phenomenon influences collectively and individuals, shaping a state for life, especially among young people [26]. Furthermore, the cinematographic habits that future teachers may have are a way of accessing knowledge of cinema and, therefore, of formation. Hence, students acquire skills not only from educational institutions, but also from other non-formal channels [27]. This double aspect generates a socio-educational approach, since it takes into account cinema as a potential educational resource and as a means of social communication. Likewise, in the Spanish context, it is particularly appropriate to analyze film habits, considering the country′s territorial distribution, which is organized around Autonomous Communities with their own political, educational and cultural identity that can be expressed in differences in students′ personal and social habits.

Based on the above, the general objective of this study is to understand the film consumption habits of teacher’s degree students in Spanish universities. The following specific objectives underlie this general objective:

1. To analyze the weekly frequency with which cinema is viewed in Spain, by Autonomous Community, by type of teacher’s degree and by type of university.
2. To examine the type of cinema usually consumed in Spain and according to the Autonomous Community, the type of teacher’s degree and the type of university.
3. To identify the devices that are usually used to watch films.

## 2. Materials and Methods

To conduct the research, a quantitative approach of a descriptive nature has been adopted with survey design [28] to collect variables from a given population in a representative manner.

### 2.1. Participants

**Figure 1:**
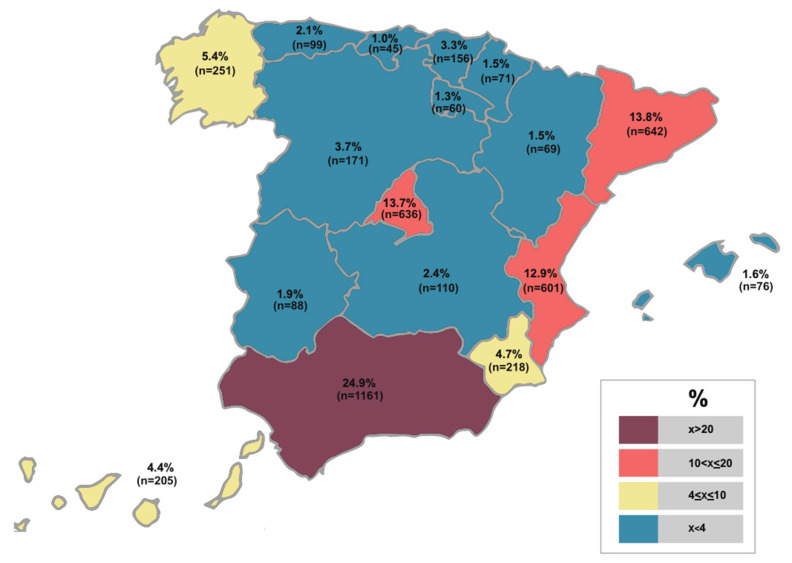
Sample percentages and frequencies by Autonomous Community.

The sample of participants, being a national study, was made up of 4659 students from all the Autonomous Communities and 58 universities, of which 39 were public and 19 privates. A technique of quota sampling was used to form the sample [29]. 15.5% (n = 720) of the participants were men and 84.5% (n = 3939) were women. They were between 18 and 66 years old and had a mean age of 22.3 (SD = 3.9). In turn, 49.0% (n = 2281) were students in the Pre-school Education Training Degree and 51.0% (n = 2378) in the Primary Education Teaching Degree. Likewise, 89.8% (n = 4183) were students from public universities and 10.2% (n = 476) from private universities. With regard to the territorial distribution, Figure 1 below shows the frequencies and percentages of the participating students according to the Autonomous Community.

If the university is attended, in Table 1, the participants are presented according to the university they belong to.

The total sample is representative of the population analyzed with a sampling error of 1.4% and a confidence level of 95.5%.

### 2.2. Instrument

The questionnaire Percepciones sobre las potencialidades del cine como recurso didáctico en las aulas de Infantil y Primaria (PECID), designed ad hoc for this research, was used for the data collection. It consists of 45 items distributed in different parts and sections. The first part of the questionnaire focuses on film consumption habits and on the training received for the didactic use of cinema. The second part of the questionnaire focuses on the perceptions about the potential of cinema as a didactic resource in Pre-school and Primary Education through a Likert-type scale, with six response categories [30]. In this study, the results on film habits are presented, which are quantified from items 6, 7 and 8 of the questionnaire. Item 6 asked about the number of movies generally watched per week. Item 7 asked about the type of cinema usually seen, being able to choose one or several options at the same time. In item 8, we asked about the device that is usually used to watch movies, being able to select one or several options at the same time. These items were subjected to expert judgment through the content validity coefficient [31]. In this sense, values higher than 0.80 were obtained, which shows a good content validity.

### 2.3. Procedure

The data collection had several phases. The public and private universities that offer the teacher′s degree were identified and then the teachers were contacted, asking for their collaboration in passing the questionnaire on to their students. Finally, data collection was conducted by completing the questionnaire during the academic year 18–19 through Google Forms. The students participated voluntarily and anonymously and had to mark a box on the questionnaire where they gave their consent to the processing of the data for this research.

### 2.4. Data Analysis

The analyses of the descriptive statistics, with the frequencies, percentages and mean were carried out through the statistical package SPSS in its version 21 (IBM Corp., Armonk, NY, USA).

## 3. Results

The results of the study, grouped according to the quantified variables, are presented below.

### 3.1. Film Viewing Frequency per Week

Regarding the number of films viewed weekly by the teacher’s degree students in Spain, the results showed that 32.8% watched one film per week and 27.0% watched two. Likewise, a significant percentage usually watched more than two films and less than six, specifically, 14.7% watched three films, 7.1% watched four and 3.7% watched five. A very small minority of students watch six, seven or more films, with 1.1%, 0.5% and 1.2% respectively. Only 12.0% say they don′t watch any films a week. On the other hand, it should be noted that the mean number of films regularly watched by degree level students is 2.09 films per week.

In order to analyze the number of films watched per week, according to the Autonomous Community, the results are presented in Table 2.

The results obtained show that the four communities with the highest mean number of film views are Extremadura, Cantabria, Aragón and Castilla y León with 2.52, 2.46, 2.42 and 2.42 respectively. In this sense, Aragón has the highest percentage of students who watch four films (15.9%) and Castilla y León has the highest percentage of students who watch five films per week (7.0%). Likewise, Extremadura also had a very high percentage watching three or more films, with 40.9%. In most Autonomous Communities, a mean of two films were watched, except in Castilla-La Mancha, Cataluña, Murcia and País Vasco, which watched a mean of one film. The communities with the highest percentage of students not watching any film per week were País Vasco (18.6%), Navarra (15.5%) and Murcia (13.8%).

Regarding the number of films watched according to the type of teacher′s degree, as can be observed in Table 3, the percentage of students in the Pre-school teacher′s degree who watched two films per week was slightly higher than that of Primary Education, 28.2% as compared with 25.9%, respectively, but the percentage of students in the Primary Education Degree, who watched four or more films per week was slightly higher. The above makes the mean almost identical.

Regarding the frequency of films by type of university, the number of students at public universities who did not watch any films per week was slightly higher, 12.1% as compared with 10.7%, and the number who watched one film per week was lower, 32.4% as compared with 36.1%, respectively. However, fewer students at private universities watched two or three films, 26.1% and 14.3% as compared with 27.1% and 14.8%, respectively. Between four and seven films, there were 12.3% of students from public universities (7.1%, 3.6%, 1.1% and 0.5%) and 12.1% from private universities (7.1%, 4.0%, 0.6% and 0.4%). In addition, 1.2% of students at public universities and 0.6% at private universities watch more than seven films per week. The mean is 2.09 in public universities and 2.02 in private universities.

### 3.2. Type of Cinema Usually Watched

The results on the type of cinema seen by students who have a weekly habit of consuming films are presented by grouping the type of cinema into three large categories, fiction, documentary and animation, and in their different combinations, starting from a dichotomous response. 57.1% of participants watched only fiction films, 1.5% watched only animated films and 0.5% watched only documentary films. If we consider the habit of watching several types of cinema at the same time, 28.9% combine fiction and animation cinema, 5.7% fiction and documentary cinema and 0.1% documentary and animation cinema. Furthermore, 6.1% habitually watched fiction, documentary and animation films at the same time.

Table 4, Table 5 and Table 6 below present the results of the type of cinema viewed, by Autonomous Community, according to whether it was fiction, documentary or animated.

As can be seen, the community with the highest percentage of students who watch only fiction films is Murcia with 64.9%, while in the rest of the community it is no less than 40.0%. For its part, documentary films are seen only exceptionally, being only in Asturias, Extremadura, Murcia and La Rioja, where more than 1% of its students watch only documentary films. On the other hand, in relation to animation cinema without combining it with other types of cinema, this is the most frequently seen in the different Autonomous Communities, although, in Canarias, Cantabria, Extremadura, Navarra and La Rioja, no student sees exclusively animation cinema. Similarly, the Communities with the highest percentage of students who watched animated films were Castilla-La Mancha, País Vasco and Andalucía, with 6.1%, 2.4% and 2.2%, respectively.

Table 7, Table 8, Table 9 and Table 10 below present the results of the combinations of the types of cinema viewed by the teacher′s degree students, based on the Autonomous Community to which they belong.

According to the data presented, 19.0% of students in each community have combined consumption habits of fiction and animation films, with Aragón and Canarias standing out with 46.8% and 40.2%, respectively. At the opposite end is La Rioja, where only 19.2% of students watch these two types of films. In turn, if we consider the combined viewing of fiction and documentary films, it is worth noting that the bulk of the student body in the different communities is between 3% and 7%. The communities with the highest percentage of students who watched these two types of films were Cataluña (8.6%) and Navarra (8.3%). In contrast, the community with the lowest number of students watching fiction and documentary films was Castilla-La Mancha. Regarding the habit of viewing documentary and animated films, only three Autonomous Communities, Cataluña, Galicia and Madrid, had students who viewed these two types of films in a combined manner. Finally, it is worth noting that all Autonomous Communities have students who regularly watch fiction, documentary and animation films at the same time. In this sense, La Rioja and Cantabria are the communities with the highest percentage of students who watch all three types of film, with 15.4% and 15.0% respectively.

Regarding the type of cinema seen according to the teacher′s degree, the results obtained are presented in Table 11.

The findings show that in the two degrees only fiction films are seen in practically the same percentage, 57.8% of students in the Primary Education degree and 56.4% in the Pre-school Education teacher degree. Nevertheless, it is worth noting that the student body of the Pre-school Education teacher degree sees a little more fiction and animation combined than the student body of the Primary Education teacher degree, specifically, 31.8% as compared with 26.2%. In contrast, the pupil in the Primary Education teacher′s degree saw slightly more fiction and documentary films combined than the pupil in the Pre-school Education teacher′s degree (7.0% as compared with 4.4%), as well as more fiction, documentary and animation films combined (6.9% as compared with 5.4%).

Regarding the type of cinema viewed, by type of university, as observed in Table 12, the students in private universities watched more fiction and documentary films than those in public universities, 9.2% as compared with 5.3%. Conversely, the students at public universities watched slightly more fiction and animation films at the same time, 29.5% as compared with the 23.8% of students at private universities. Finally, it is worth noting that no participating students from private universities watch both documentary and animated films.

### 3.3. Devices to Watch Movies Regularly

Regarding the devices used to watch movies by the teacher′s degree students who watch movies weekly, the results of the answers from the multiple choice are presented (Table 13).

The results showed an outstanding use of the television (36.6%) and the computer (34.9%) as the most common devices for watching movies, with the cinema projector being the third most used medium, although with a considerably lower percentage (17.1%).

## 4. Discussion

The aim of this work has been to know the film habits of the teacher′s degree students in Spain. The findings revealed that 88% are regular film consumers per week. In this sense, 59.8% usually watch one or two films, reaching 74.5% if we add the students who watch three films a week. If we look at the overall mean, this is two films per week. These results coincide with those obtained by [32], who analyzed the film consumption habits of Spanish and Latin American university students and reflected a regular, albeit moderate, consumption of films, that is, not daily. If we look at the results obtained according to the Autonomous Community, students in Extremadura and Cantabria have the highest film viewing habits. In this sense, the students from Extremadura and Cantabria who watch three or more films a week rise to 40.9% and 33.3%, respectively. On the opposite side are País Vasco, Navarra and Murcia, which have a percentage of more than 13% of students who do not watch any films per week. Ref. [25] found that a higher frequency of film viewing leads to a more positive perception of the potential of cinema as a didactic resource among teacher′s degree students. These differences manifest themselves per student and per region and have their effect on the future application of cinema in the classroom. If the student body is compared according to the type of teacher′s degree and the type of university, the number of films watched per week is very similar, so it can be said that this is not a differentiating factor. In this sense, the student body of the two grades watched two movies per week, although the student body of the Pre-school teacher′s degree saw a slightly higher percentage (28.1%) of movies per week, while the students of the Primary Education teacher′s degree watched a little more usually one movie per week (33.2%). In turn, the student body at public universities who did not watch any films per week was slightly higher (12.1%) than that belonging to private universities. These similarities coincide with the analysis of [32], which found similar frequencies of film viewing by students from different undergraduate studies and universities.

As regards the type of cinema usually seen, the study has shown a habit of cinema where fiction cinema clearly prevails, as 57.13% of those surveyed see only fiction cinema and when several types of cinema are usually combined, the most common combinations are always with fiction cinema, as is the case of those who see fiction cinema and animation cinema, which is 28.94%. These findings are similar to the results obtained by [33,34,35], which show that audiovisual consumption is essentially associated with fiction and its various sub-genres. In this sense, animation and documentary remain the most minority genres, especially the latter.

If we look at the Autonomous Communities, Murcia is the community that watches the most fiction films exclusively (64.9%), while documentaries are not watched exclusively in any autonomy more than 2%. The community that watches more animated films exclusively is Castilla-La Mancha (6.1%), and it is clear that the communities that watch more animated films only are those that watch less documentary films, as is the case of La Rioja and Extremadura, in line with the contrast between these two types of cinema, since animation is far from reality and the documentary aims at an objective register or sincere reconstruction [36]. This fact is reproduced when watching animation and documentary at the same time, since, in fourteen Autonomous Communities, there are no students with this filmmaking habit. Aragón, on the other hand, is the Autonomous Community whose students watch the most fiction and animation films together (46.8%), Cataluña, where they watch the most fiction and documentary films together, and La Rioja and Cantabria, whose students most regularly watch fiction, documentary and animation films together. The data obtained are relevant due to the fact that, as they have stated [25], having a habit of consuming only fiction films determines a less positive perception of cinema as a didactic resource. On the other hand, the fact that students usually only watch documentaries on several types of films at the same time has a positive influence on their perception of the educational possibilities of cinema. Considering the type of teacher′s degree and the type of university, as was the case with the number of films viewed per week, the results reflected that the habits regarding the type of cinema viewed were almost identical, once again confirming that it was not a variable that originated relevant differences, in line with the findings of [32].

With regard to movie viewing devices, it has been reflected that 71.5% of students have as their preferred media the television and the computer, the former being the most used by 36.6%. These results reflect the diversification of film consumption devices, above the original mean of watching films, which was the cinema, in a “multi-screen society” [37] (p. 22) and technological variety, in line with the studies of [38,39,40]. Similarly, the studies of [41,42,43,44] confirm the coexistence in audiovisual consumption of young Spanish people between television and the growing space of the Internet through multiple devices, and, according to [42], the preference for cinema consumption on television increases among people over 18. This fact can be said to affect the way we approach the reality of cinema and the experience that cinema can generate, being more individualistic and fragmented. In this sense, the results of the study carried out by [45] shows that cinema attendance among the Spanish population has decreased considerably, with 53% never or almost never going to the cinema, although among the youngest people, attendance is 5% higher than among the rest of the population. In short, these data, together with those obtained from the frequency of film viewing, show that cinema has a penetration in the population studied, but increasingly from the home and less and less from the cinema. These data are reinforced by the studies of [46,47], which shows that young Spanish people and university students spend more time-consuming fictional television series using a computer, to the detriment of other audiovisual content such as films.

## 5. Conclusions

Based on the proposed aims and the findings, the following conclusions are indicated:

1. Most students in the teacher′s degree have a habit of watching two films a week.
2. The type of cinema that is usually seen is fiction cinema, watching it exclusively or combining it with other types of cinema to a lesser extent.
3. The most common means of watching movies are television and the computer.

As a limitation of the study, it is worth mentioning the fact that five private Spanish universities refused to collaborate in the study. This meant that a larger and more balanced sample could not be obtained, and that students from all universities participated.

The study presented is a pioneer in analyzing this theme in Spain. The confirmation that futures teachers are habitual consumers of cinema should serve to promote specific training in the teacher′s degree that contributes to an optimal application of cinema as a didactic resource, complementing the knowledge that the students have of cinema outside the university. If cinema is a resource with educational potential which does not require a very expensive economic investment and besides is a popular medium among future teachers, then the possibilities of implementing training in the didactic use of cinema are greater. This could be done through a specific subject or in a transversal way in each area of knowledge. In this sense, a specific subject should be included that addresses training in audiovisual resources, such as film, for educational use. This subject should be addressed by going beyond technical approaches that reduce cinema to a mere technological instrument, giving priority to reflective approaches, critical analysis and knowledge creation. Its contents should address didactic training for the educational use of cinema. In addition to this, cinema must be approached in this subject as an audiovisual language and a means of artistic expression. Moreover, cinema could be a resource to work in a transversal way in the agenda of subjects, in order to explore its educational possibilities in different knowledge areas. In this sense, priority should be given to training in the creation of activities through cinema. Furthermore, teachers of Teacher Training degree programs should carry out activities with students using film. Training for the didactic use of cinema should be extended to all Autonomous Communities, although with more intensity in those whose students have a lesser habit of watching films. Furthermore, training should be developed with the use of different types of cinema, filling one of the gaps detected in the cinematographic habits of students in the teacher′s degree. The educational possibilities of documentary films [48] and animation films [49], not only fiction films, have been highlighted.

On the other hand, in order to encourage film consumption habits in line with educational objectives, it is appropriate for education faculties should conduct training initiatives to promote cinema with special cultural and educational value, that can contribute to better teacher performance in the future. Consequently, film screenings and activities should be organized to make visible to students a cinema that enriches their cultural background and promotes a critical and reflective vision of cinematographic works to develop mature and responsible viewers. In this line, students should also be encouraged to get used to seeing a greater variety of types of cinema, beyond fiction films, promoting, for example, documentary films that have a lesser commercial diffusion. Furthermore, we should also encourage the habit of going to the cinema, to recover the original experience of cinema in all its breadth. This could be done through collaboration agreements between universities and cinemas, which would allow greater access to theatres for educational purposes. All of that, with the purpose of favoring the personal enrichment of the students and promoting a transference to the social-educational context.

## Figures and Tables

**Table 1:** Participant sample according to the university.

University	*f*	%
University of A Coruña	119	2.6
University of Alcalá	166	3.6
Alfonso X El Sabio University	3	0.1
University of Alicante	196	4.2
University of Almería	23	0.5
Nebrija University	19	0.4
Autonomous University of Barcelona	73	1.6
Autonomous University of Madrid	154	3.3
University of Barcelona	64	1.4
University of Burgos	24	0.5
University of Cádiz	155	3.3
Camilo José Cela University	24	0.5
University of Cantabria	41	0.9
CEU Cardenal Herrera University	35	0.8
University of Castilla La Mancha	110	2.4
Catholic University San Antonio	33	0.7
Catholic University Santa Teresa de Jesús de Ávila	2	0.0
Valencia Catholic University San Vicente Mártir	9	0.2
Complutense University of Madrid	45	1.0
University of Córdoba	224	4.8
University of Deusto	13	0.3
European University of the Atlantic	4	0.1
University of Extremadura	88	1.9
Francisco de Vitoria University	13	0.3
University of Girona	158	3.4
University of Granada	116	2.5
University of Huelva	101	2.2
University of the Islas Baleares	76	1.6
International University of Cataluña	8	0.2
Jaume I University	223	4.8
University of Jaén	25	0.5
University of La Laguna	88	1.9
University of La Rioja	60	1.3
University of Las Palmas de Gran Canaria	117	2.5
University of León	27	0.6
University of Lleida	111	2.4
Loyola University Andalucía	30	0.6
Mondragón University	70	1.5
University of Murcia	185	4.0
University of Málaga	186	4.0
University of Navarra	23	0.5
University of Oviedo	99	2.1
University of País Vasco	73	1.6
Comillas Pontifical University	32	0.7
Pontifical University of Salamanca	33	0.7
Public University of Navarra	48	1.0
Ramon Llull University	96	2.1
Rey Juan Carlos University	180	3.9
Rovira i Virgili University	118	2.5
University of Salamanca	54	1.2
San Jorge University	15	0.3
University of Santiago de Compostela	35	0.8
University of Sevilla	301	6.5
University of Valladolid	31	0.7
University of Valencia	138	3.0
University of Vic	14	0.3
University of Vigo	97	2.1
University of Zaragoza	54	1.2
Total	4659	100.0

**Table 2:** Percentages and frequencies of the number of films viewed, according to the Autonomous Community.

Autonomous Community	Number of Films per Week											
		No One	1	2	3	4	5	6	7	+7	Total	M
Andalucía	*f*	126	389	310	179	86	41	10	6	14	1161	2.09
	*% AC.*	10.9	33.5	26.7	15.4	7.4	3.5	0.9	0.5	1.2	100.0	
	*%SPN.*	2.7	8.3	6.7	3.8	1.8	0.9	0.2	0.1	0.3	24.9	
Aragón	*f*	7	15	20	13	11	0	0	2	1	69	2.42
	*% AC.*	10.1	21.7	29.0	18.8	15.9	0.0	0.0	2.9	1.4	100.0	
	*%SPN.*	0.2	0.3	0.4	0.3	0.2	0.0	0.0	0.0	0.0	1.5	
Asturias	*f*	12	30	26	18	9	1	1	0	2	99	2.13
	*% AC.*	12.1	30.3	26.3	18.2	9.1	1.0	1.0	0.0	2.0	100.0	
	*%SPN.*	0.3	0.6	0.6	0.4	0.2	0.0	0.0	0.0	0.0	2.1	
Islas Baleares	*f*	5	24	25	11	8	2	0	0	1	76	2.13
	*% AC.*	6.6	31.6	32.9	14.5	10.5	2.6	0.0	0.0	1.3	100.0	
	*%SPN.*	0.1	0.5	0.5	0.2	0.2	0.0	0.0	0.0	0.0	1.6	
Canarias	*f*	21	52	58	37	14	12	3	2	6	205	2.41
	*% AC.*	10.2	25.4	28.3	18.0	6.8	5.9	1.5	1.0	2.9	100.0	
	*%SPN.*	0.5	1.1	1.2	0.8	0.3	0.3	0.1	0.0	0.1	4.4	
Cantabria	*f*	5	9	16	7	3	2	1	0	2	45	2.46
	*% AC.*	11.1	20.0	35.6	15.6	6.7	4.4	2.2	0.0	4.4	100.0	
	*%SPN.*	0.1	0.2	0.3	0.2	0.1	0.0	0.0	0.0	0.0	1.0	
Castilla-La Mancha	*f*	11	42	33	17	3	4	0	0	0	110	1.83
	*% AC.*	10.0	38.2	30.0	15.5	2.7	3.6	0.0	0.0	0.0	100.0	
	*%SPN.*	0.2	0.9	0.7	0.4	0.1	0.1	0.0	0.0	0.0	2.4	
Castilla y León	*f*	19	42	46	28	18	12	0	2	4	171	2.42
	*% AC.*	11.1	24.6	26.9	16.4	10.5	7.0	0.0	1.2	2.3	100.0	
	*%SPN.*	0.4	0.9	1.0	0.6	0.4	0.3	0.0	0.0	0.1	3.7	
Cataluña	*f*	87	252	155	80	39	17	6	1	5	642	1.88
	*%AC.*	13.6	39.3	24.1	12.5	6.1	2.6	0.9	0.2	0.8	100.0	
	*%SPN.*	1.9	5.4	3.3	1.7	0.8	0.4	0.1	0.0	0.1	13.8	
Comunidad Valenciana	*f*	72	192	173	84	38	25	8	4	5	601	2.08
	*% AC.*	12.0	31.9	28.8	14.0	6.3	4.2	1.3	0.7	0.8	100.0	
	*%SPN.*	1.5	4.1	3.7	1.8	0.8	0.5	0.2	0.1	0.1	12.8	
Extremadura	*f*	9	19	24	18	8	4	4	0	2	88	2.52
	*% AC.*	10.2	21.6	27.3	20.5	9.1	4.5	4.5	0.0	2.3	100.0	
	*%SPN.*	0.2	0.4	0.5	0.4	0.2	0.1	0.1	0.0	0.0	1.9	
Galicia	*f*	32	74	64	44	16	11	5	0	5	251	2.21
	*% AC.*	12.7	29.5	25.5	17.5	6.4	4.4	2.0	0.0	2.0	100.0	
	*%SPN.*	0.7	1.6	1.4	0.9	0.3	0.2	0.1	0.0	0.1	5.4	
Madrid	*f*	74	208	173	90	45	29	10	1	6	636	2.10
	*% AC.*	11.6	32.7	27.2	14.2	7.1	4.6	1.6	0.2	0.9	100.0	
	*%SPN.*	1.6	4.5	3.7	1.9	1.0	0.6	0.2	0.0	0.1	13.7	
Murcia	*f*	30	81	62	24	13	5	2	0	1	218	1.85
	*% AC.*	13.8	37.2	28.4	11.0	6.0	2.3	0.9	0.0	0.5	100.0	
	*%SPN.*	0.6	1.7	1.3	0.5	0.3	0.1	0.0	0.0	0.0	4.7	
Navarra	*f*	11	22	19	10	5	1	1	2	0	71	2.05
	*% AC.*	15.5	31.0	26.8	14.1	7.0	1.4	1.4	2.8	0.0	100.0	
	*%SPN.*	0.2	0.5	0.4	0.2	0.1	0.0	0.0	0.0	0.0	1.5	
País Vasco	*f*	29	56	39	17	10	4	0	1	0	156	1.80
	*% AC.*	18.6	35.9	25.0	10.9	6.4	2.6	0.0	0.6	0.0	100.0	
	*%SPN.*	0.6	1.2	0.8	0.4	0.2	0.1	0.0	0.0	0.0	3.3	
La Rioja	*f*	8	19	16	8	6	1	0	2	0	60	2.10
	*% AC.*	13.3	31.7	26.7	13.3	10.0	1.7	0.0	3.3	0.0	100.0	
	*%SPN.*	0.2	0.4	0.3	0.2	0.1	0.0	0.0	0.0	0.0	1.3	
Total	*f*										4659	
	*%SPN.*										100.0	

**Table 3:** Number of films watched per week by the teacher′s degree students, according to the type of degree.

Number of Films	Teacher’s Degree in Pre-School Education			Teacher’s Degree in Primary Education			Total	
	*f*	*%Pre.*	Total %	*f*	*%Pri.*	Total %	*f*	*%Spain*
No one	266	11.7	5.7	292	12.3	6.3	558	12.0
1	737	32.3	15.8	789	33.2	16.9	1526	32.8
2	642	28.1	13.8	617	25.9	27.0	1259	27.0
3	334	14.6	7.2	351	14.8	7.5	685	14.7
4	162	7.1	3.5	170	7.1	3.6	332	7.1
5	80	3.5	1.7	91	3.8	2.0	171	3.7
6	27	1.2	1.6	24	1.0	0.5	51	1.1
7	9	0.4	0.2	14	0.6	0.3	23	0.5
+7	24	1.1	0.5	30	1.3	1.2	54	1.2
Total	2281	100.0	49.0	2378	100.0	51.0	4659	100.0
Mean	2.08			2.09				

**Table 4:** Percentages and frequencies of fiction films seen by teacher′s degree students, according to the Autonomous Community.

FICTION									
Autonomous Community	YES			NO			Total		
	*f*	*%AC.*	*%Spn.*	*f*	*%AC.*	*%Spn.*	*f*	*%AC.*	*%Spn.*
Andalucía	619	59.8	15.1	416	40.2	10.1	1035	100.0	25.2
Aragón	25	40.3	0.6	37	59.7	0.9	62	100.0	1.5
Asturias	47	50.4	1.1	40	46.0	1.0	87	100.0	2.1
Islas Baleares	40	56.3	1.0	31	43.7	0.8	71	100.0	1.7
Canarias	83	45.1	2.0	101	54.9	2.5	184	100.0	4.5
Cantabria	20	50.0	0.5	20	50.0	0.5	40	100.0	1.0
Castilla-La Mancha	61	61.6	1.5	38	38.4	0.9	99	100.0	2.4
Castilla y León	82	53.9	2.0	70	46.1	1.7	152	100.0	3.7
Cataluña	331	59.6	8.1	224	40.4	5.5	555	100.0	13.5
Comunidad Valenciana	320	60.5	7.8	209	39.5	5.1	529	100.0	12.9
Extremadura	43	54.4	1.0	36	45.6	0.9	79	100.0	1.9
Galicia	121	55.3	3.0	98	44.7	2.4	219	100.0	5.3
Madrid	293	52.1	7.1	269	47.9	6.6	562	100.0	13.7
Murcia	122	64.9	3.0	66	35.1	1.6	188	100.0	4.6
Navarra	28	46.7	0.7	32	53.3	0.8	60	100.0	1.5
País Vasco	77	60.6	1.9	50	39.4	1.2	127	100.0	3.1
La Rioja	31	59.6	0.8	21	40.4	0.5	52	100.0	1.3
Total							4101		100.0

**Table 5:** Percentages and frequencies of documentary cinema seen by teacher′s degree students, according to the Autonomous Community.

DOCUMENTARY									
Autonomous Community	YES			NO			Total		
	*f*	*%AC.*	*%Spn.*	*f*	*%AC.*	*%Spn.*	*f*	*%AC.*	*%Spn.*
Andalucía	4	0.4	0.1	1031	99.6	25.1	1035	100.0	25.2
Aragón	0	0.0	0.0	62	100.0	1.5	62	100.0	1.5
Asturias	1	1.1	0.0	86	98.9	2.1	87	100.0	2.1
Islas Baleares	0	0.0	0.0	71	100.0	1.7	71	100.0	1.7
Canarias	0	0.0	0.0	184	100.0	4.5	184	100.0	4.5
Cantabria	0	0.0	0.0	40	100.0	0.0	40	100.0	1.0
Castilla-La Mancha	1	1.0	0.0	98	99.0	99.0	99	100.0	2.4
Castilla y León	0	0.0	0.0	152	100.0	3.7	152	100.0	3.7
Cataluña	2	0.4	0.0	553	99.6	13.5	555	100.0	13.5
Comunidad Valenciana	2	0.4	0.0	527	99.6	12.9	529	100.0	12.9
Extremadura	1	1.3	0.0	78	98.7	1.9	79	100.0	1.9
Galicia	1	0.5	0.0	218	99.5	5.3	219	100.0	5.3
Madrid	4	0.7	0.1	558	99.3	13.6	562	100.0	13.7
Murcia	2	1.1	0.0	186	98.9	4.5	188	100.0	4.6
Navarra	0	0.0	0.0	60	100.0	1.5	60	100.0	1.5
País Vasco	1	0.8	0.0	126	99.2	3.1	127	100.0	3.1
La Rioja	1	1.9	0.0	51	98.1	1.2	52	100.0	1.3
Total							4101		100.0

**Table 6:** Percentages and frequencies of animation films seen by teacher′s degree students, according to the Autonomous Community.

ANIMATION									
Autonomous Community	YES			NO			Total		
	*f*	*%AC.*	*%Spn.*	*f*	*%AC.*	*%Spn.*	*f*	*%AC.*	*%Spn.*
Andalucía	23	2.2	0.6	1012	97.8	24.7	1035	100.0	25.2
Aragón	1	1.6	0.0	61	98.4	1.5	62	100.0	1.5
Asturias	1	1.1	0.0	86	98.9	2.1	87	100.0	2.1
Islas Baleares	1	1.4	0.0	70	98.6	1.7	71	100.0	1.7
Canarias	0	0.0	0.0	184	100.0	4.5	184	100.0	4.5
Cantabria	0	0.0	0.0	40	100.0	1.0	40	100.0	1.0
Castilla-La Mancha	6	6.1	0.1	93	93.9	2.3	99	100.0	2.4
Castilla y León	2	1.3	0.0	150	98.7	3.7	152	100.0	3.7
Cataluña	4	0.7	0.1	551	99.3	13.4	555	100.0	13.5
Comunidad Valenciana	5	0.9	0.1	524	99.1	12.8	529	100.0	12.9
Extremadura	0	0.0	0.0	79	100.0	1.9	79	100.0	1.9
Galicia	1	0.5	0.0	218	99.5	5.3	219	100.0	5.3
Madrid	10	1.8	0.2	552	98.2	13.5	562	100.0	13.7
Murcia	3	1.6	0.1	185	98.4	4.5	188	100.0	4.6
Navarra	0	0.0	0.0	60	100.0	1.5	60	100.0	1.5
País Vasco	3	2.4	0.1	124	97.6	3.0	127	100.0	3.1
La Rioja	0	0.0	0.0	52	100.0	1.3	52	100.0	1.3
Total							4101		100.0

**Table 7:** Percentages and frequencies of fiction and documentary films seen by teacher′s degree students, according to the Autonomous Community.

FICTION + DOCUMENTARY									
Autonomous Community	YES			NO			Total		
	*f*	*%AC.*	*%Spn.*	*f*	*%AC.*	*%Spn.*	*f*	*%AC.*	*%Spn.*
Andalucía	44	4.3	1.1	991	95.7	24.2	1035	100.0	25.2
Aragón	3	4.8	0.1	59	95.2	1.4	62	100.0	1.5
Asturias	3	3.4	0.1	84	96.6	2.0	87	100.0	2.1
Islas Baleares	4	5.6	0.1	67	94.4	1.6	71	100.0	1.7
Canarias	10	5.4	0.2	174	94.6	4.2	184	100.0	4.5
Cantabria	3	7.5	0.1	37	92.5	0.9	40	100.0	1.0
Castilla-La Mancha	2	2.0	0.0	97	98.0	2.4	99	100.0	2.4
Castilla y León	6	3.9	0.1	146	96.1	3.6	152	100.0	3.7
Cataluña	48	8.6	1.2	507	91.4	12.4	555	100.0	13.5
Comunidad Valenciana	36	6.8	0.9	493	93.2	12.0	529	100.0	12.9
Extremadura	5	6.3	0.1	74	93.7	1.8	79	100.0	1.9
Galicia	13	5.9	0.3	206	94.1	5.0	219	100.0	5.3
Madrid	33	5.9	0.8	529	94.1	12.9	562	100.0	13.7
Murcia	8	4.3	0.2	180	95.7	4.4	188	100.0	4.6
Navarra	5	8.3	0.1	55	91.7	1.3	60	100.0	1.5
País Vasco	9	7.1	0.2	118	92.9	2.9	127	100.0	3.1
La Rioja	2	3.8	0.0	50	96.2	1.2	52	100.0	1.3
Total							4101		100.0

**Table 8:** Percentages and frequencies of fiction and animation films seen by teacher′s degree students, according to the Autonomous Community.

FICTION + ANIMATION									
Autonomous Community	YES			NO			Total		
	*f*	*%AC.*	*%Spn.*	*f*	*%AC.*	*%Spn.*	*f*	*%AC.*	*%Spn.*
Andalucía	297	28.7	7.2	738	71.3	18.0	1035	100.0	25.2
Aragón	29	46.8	0.7	33	53.2	0.8	62	100.0	1.5
Asturias	31	35.6	0.8	56	64.4	1.4	87	100.0	2.1
Islas Baleares	19	26.8	0.5	52	73.2	1.3	71	100.0	1.7
Canarias	74	40.2	1.8	110	59.8	2.7	184	100.0	4.5
Cantabria	11	27.5	0.3	29	72.5	0.7	40	100.0	1.0
Castilla-La Mancha	27	27.3	0.7	72	72.7	1.8	99	100.0	2.4
Castilla y León	47	30.9	1.1	105	69.1	2.6	152	100.0	3.7
Cataluña	130	23.4	3.2	425	76.6	10.4	555	100.0	13.5
Comunidad Valenciana	146	27.6	3.6	383	72.4	9.3	529	100.0	12.9
Extremadura	26	32.9	0.6	53	67.1	1.3	79	100.0	1.9
Galicia	66	30.1	1.6	153	69.9	3.7	219	100.0	5.3
Madrid	180	32.0	4.4	382	68.0	9.3	562	100.0	13.7
Murcia	45	23.9	1.1	143	76.1	3.5	188	100.0	4.6
Navarra	21	35.0	0.5	39	65.0	1.0	60	100.0	1.5
País Vasco	28	22.0	0.7	99	78.0	2.4	127	100.0	3.1
La Rioja	10	19.2	0.2	42	80.8	1.0	52	100.0	1.3
Total							4101		100.0

**Table 9:** Percentages and frequencies of documentary and animation films seen by teacher′s degree students, according to the Autonomous Community.

DOCUMENTARY + ANIMATION									
Autonomous Community	YES			NO			Total		
	*f*	*%AC.*	*%Spn.*	*f*	*%AC.*	*%Spn.*	*f*	*%AC.*	*%Spn.*
Andalucía	0	0.0	0.0	1035	100.0	25.2	1035	100.0	25.2
Aragón	0	0.0	0.0	62	100.0	1.5	62	100.0	1.5
Asturias	0	0.0	0.0	87	100.0	2.1	87	100.0	2.1
Islas Baleares	0	0.0	0.0	71	100.0	1.7	71	100.0	1.7
Canarias	0	0.0	0.0	184	100.0	4.5	184	100.0	4.5
Cantabria	0	0.0	0.0	40	100.0	1.0	40	100.0	1.0
Castilla-La Mancha	0	0.0	0.0	99	100.0	2.4	99	100.0	2.4
Castilla y León	0	0.0	0.0	152	100.0	3.7	152	100.0	3.7
Cataluña	3	0.5	0.1	552	99.5	13.5	555	100.0	13.5
Comunidad Valenciana	0	0.0	0.0	529	100.0	12.9	529	100.0	12.9
Extremadura	0	0.0	1.9	79	100.0	1.9	79	100.0	1.9
Galicia	2	0.9	0.0	217	99.1	5.3	219	100.0	5.3
Madrid	1	0.2	0.0	561	99.8	13.7	562	100.0	13.7
Murcia	0	0.0	0.0	188	100.0	4.6	188	100.0	4.6
Navarra	0	0.0	0.0	60	100.0	1.5	60	100.0	1.5
País Vasco	0	0.0	0.0	127	100.0	3.1	127	100.0	3.1
La Rioja	0	0.0	0.0	52	100.0	1.3	52	100.0	1.3
Total							4101		100.0

**Table 10:** Percentages and frequencies of fiction, documentary and animation films seen by teacher′s degree students, according to the Autonomous Community.

FICTION + DOCUMENTARY + ANIMATION									
Autonomous Community	YES			NO			Total		
	*f*	*%AC.*	*%Spn.*	*f*	*%AC.*	*%Spn.*	*f*	*%AC.*	*%Spn.*
Andalucía	48	4.6	1.2	987	95.4	24.1	1035	100.0	25.2
Aragón	4	6.5	0.1	58	93.5	1.4	62	100.0	1.5
Asturias	4	4.6	0.1	83	95.4	2.0	87	100.0	2.1
Islas Baleares	7	9.9	0.2	64	90.1	1.6	71	100.0	1.7
Canarias	17	9.2	0.4	167	90.8	4.1	184	100.0	4.5
Cantabria	6	15.0	0.1	34	85.0	0.8	40	100.0	1.0
Castilla-La Mancha	2	2.0	0.0	97	98.0	2.4	99	100.0	2.4
Castilla y León	15	9.9	0.4	137	90.1	3.3	152	100.0	3.7
Cataluña	37	6.7	0.9	518	93.3	12.6	555	100.0	13.5
Comunidad Valenciana	20	3.8	0.5	509	96.2	12.4	522	100.0	12.9
Extremadura	4	5.1	0.1	75	94.9	1.8	79	100.0	1.9
Galicia	15	6.8	0.4	204	93.2	5.0	219	100.0	5.3
Madrid	41	7.3	1.0	521	92.7	12.7	562	100.0	13.7
Murcia	8	4.3	0.2	180	95.7	4.4	188	100.0	4.6
Navarra	6	10.0	0.1	54	90.0	1.3	60	100.0	1.5
País Vasco	9	7.1	0.2	118	92.9	2.9	127	100.0	3.1
La Rioja	8	15.4	0.2	44	84.6	1.1	52	100.0	1.3
Total							4101		100.0

**Table 11:** Percentages and frequencies of the type of cinema seen by the students, according to the type of teacher′s degree.

Type of Cinema	Teacher’s Degree in Pre-School Education									Teacher’s Degree in Primary Education										
	YES			NO			Total Pre-school Degree			YES			NO			Total Primary Degree			Total	
	*f*	*%AC.*	*%Spn.*	*f*	*%AC.*	*%Spn.*	*f*	*% AC.*	*%Spn.*	*f*	*%AC.*	*%Spn.*	*f*	*%AC.*	*%Spn.*	*f*	*%AC.*	*%Spn.*	*f*	%
Fic.	1137	56.4	27.7	878	43.6	21.4	2015	100.0	49.1	1206	57.8	29.4	880	42.2	21.5	2086	100.0	50.9	4101	100.0
Doc.	8	0.4	0.2	2007	99.6	48.9	2015	100.0	49.1	12	0.6	0.3	2074	99.4	50.6	2086	100.0	50.9	4101	100.0
Ani.	31	1.5	0.8	1984	98.5	48.4	2015	100.0	49.1	29	1.4	0.7	2057	98.6	50.2	2086	100.0	50.9	4101	100.0
Fic. + Doc.	88	4.4	2.1	1927	95.6	47.0	2015	100.0	49.1	146	7.0	3.6	1940	93.0	47.3	2086	100.0	50.9	4101	100.0
Fic. + Ani.	641	31.8	15.6	1374	68.2	33.5	2015	100.0	49.1	546	26.2	13.3	1540	73.8	37.6	2086	100.0	50.9	4101	100.0
Doc. + Ani.	2	0.1	0.0	2013	99.9	49.1	2015	100.0	49.1	4	0.2	0.1	2082	99.8	50.8	2086	100.0	50.9	4101	100.0
Fic. + Doc. + Ani.	108	5.4	2.6	1907	94.6	46.5	2015	100.0	49.1	143	6.9	3.5	1943	93.1	47.4	2086	100.0	50.9	4101	100.0

Note: Fic. = Fiction; Doc. = Documentary; Ani. = Animation.

**Table 12:** Percentages and frequencies of devices used to watch movies by teacher’s degree students.

Device Type	*f*	%
Cinema Projector	1412	17.1
Television	3019	36.6
Computer	2875	34.9
Mobile	418	5.1
Tablet	510	6.2
Home Projector	5	0.1
Netflix	5	0.1
Movistar+	1	0.0
Playstation	2	0.0
Chromecast	2	0.0
Total	8249	100.0

**Table 13:** Percentages and frequencies of the type of cinema viewed by the teacher′s degree students, according to type of university.

Type of Cinema	Public University									Private University										
	YES			NO			Total Public University			YES			NO			Total Private University			Total	
	*f*	*%AC.*	*%Spn.*	*f*	*%AC.*	*%Spn.*	*f*	*%AC.*	*%Spn.*	*f*	*%AC.*	*%Spn.*	*f*	*%AC.*	*%Spn.*	*f*	*%AC.*	*%Spn.*	*f*	%
Fic.	2093	56.9	51.0	1583	43.1	38.6	3676	100.0	89.6	250	58.8	6.1	175	41.2	4.3	425	100.0	10.4	4101	100.0
Doc.	19	0.5	0.5	3657	99.5	89.2	3676	100.0	89.6	1	0.2	0.0	424	99.8	10.3	425	100.0	10.4	4101	100.0
Ani.	55	1.5	1.3	3621	98.5	88.3	3676	100.0	89.6	5	1.2	0.1	420	98.8	10.2	425	100.0	10.4	4101	100.0
Fic. + Doc.	198	5.3	4.8	3481	94.7	84.9	3676	100.0	89.6	39	9.2	1.0	386	90.8	9.4	425	100.0	10.4	4101	100.0
Fic. + Ani.	1086	29.5	26.5	2590	70.5	63.2	3676	100.0	89.6	101	23.8	2.5	324	76.2	7.9	425	100.0	10.4	4101	100.0
Doc. + Ani.	6	0.2	0.1	3670	99.8	89.5	3676	100.0	89.6	0	0.0	0.0	425	100.0	10.4	425	100.0	10.4	4101	100.0
Fic. + Doc. + Ani.	222	6.0	5.4	3454	94.0	84.2	3676	100.0	89.6	29	6.8	0.7	396	93.2	9.7	425	100.0	10.4	4101	100.0

Note: Fic. = Fiction; Doc. = Documentary; Ani. = Animation.

## References

[1] Amar V. (2009). El cine en la encrucijada de la educación y el conocimiento. Enl@ce.

[2] Burn A., Reid M. (2012). Screening Literacy: Reflecting on Models of Film Education in Europe. Nord. J. Digit. Lit..

[3] Martínez-Salanova E. (2003). El valor del cine para aprender y enseñar. Comunicar.

[4] Martínez-Salanova E. (2017). Educomunicación. El cine para educar. Los pintores en el cine (I). Aularia.

[5] Martínez-Salanova E. (2019). El periodismo de investigación en el cine. Aularia.

[6] Lara F., Ruiz M., Tarín M. (2019). Cine Y Educación.

[7] González M.C., Martínez E., Pereira C. (2018). Cine de animación y educación. Modelos de películas de animación y sus virtualidades educativas. Reladei.

[8] Gauld C., Wathelet M., Medjkane F., Pauwels N., Bougerol T., Notredame C.E. (2019). Construction and Validation of an Analytical Grid about Video Representations of Suicide (“MoVIES”). Int. J. Environ. Res. Public Health.

[9] Cuevas J. (2020). Imaginarios sociales sobre uso de tecnología y relaciones interpersonales en jóvenes universitarios a través del cine de ficción como recurso didáctico. Sophia.

[10] Soler L. (2005). El documental: Gritos y susurros. Trípodos.

[11] Grande-López V., Pérez A. (2016). Personajes de animación con discapacidad, a través de una perspectiva educativa. Creat. Soc..

[12] Perea K. (2018). Touching Queerness in Disney Films Dumbo and Lilo & Stitch. Soc. Sci..

[13] Martínez-Salanova E. (2002). Aprender con el Cine, Aprender de Película: Una Visión Didáctica para Aprender e Investigar con el Cine.

[14] Amar V. (2009). El Cine y Otras Miradas. Contribuciones a la Educación y a la Cultura Audiovisual.

[15] Bergala A. (2007). La Hipótesis del Cine: Pequeño Tratado Sobre la Transmisión del Cine en la Escuela y Fuera de Ella.

[16] Kadivar M., Mafinejad M.K., Bazzaz J.T., Mirzazadeh A., Jannat Z. (2018). Cinemedicine: Using movies to improve students’ understanding of psychosocial aspects of medicine. Ann. Med. Surg..

[17] Ambròs A., Breu R. (2017). Cine y Educación: El Cine en el Aula de Primaria y Secundaria.

[18] Sánchez W., Uribe A., Restrepo J. (2019). El cine: Una alternativa de aprendizaje. Tri. Cienc. Tec. Soc..

[19] Ortega J.A., Pérez A. (2013). El cine digital en la formación inicial del profesorado: Una experiencia innovadora realizada en la Universidad de Granada. Educación XXI.

[20] Guichot-Reina V., Rueda J.D. (2010). El cine como mirada a nuestro pasado educativo reciente: Los días del pasado”, recurso para analizar la educación en el periodo franquista. El Cine Como Recurso Metodológico en la Docencia de Historia de la Educación.

[21] Muñoz J.M. (2001). El cine como recurso didáctico. Campo Abierto.

[22] Mujika J., Gaintza Z. (2020). El cine como herramienta didáctica en la escuela inclusiva. Didacticae.

[23] Nigro P.M. (2004). La educación para los medios. Educ. Educ..

[24] Peñalver T. (2015). El cine como recurso didáctico: Una propuesta de programación didáctica. Edetania.

[25] Lorenzo-Lledó A., Lledó A., Lorenzo G. (2020). Cinema as a Transmitter of Content: Perceptions of Future Spanish Teachers for Motivating Learning. Sustainability.

[26] Rengifo Y. (2018). The cinema in communicative environments. Resilient Adolescence. Escribanía.

[27] Harris J., Wihak C. (2018). The recognition of Non-Formal Education in Higher Education: Where Are We Now, and Are We Learning from Experience?. Int. J. E-Learn. Distance Educ..

[28] Arnau J. (1997). Model General D’investigación Psicológica.

[29] Bisquerra R. (2014). Metodología De La Investigación Educativa.

[30] Lorenzo-Lledó A. (2019). Percepciones del Alumnado del Grado de Maestro en Universidades Españolas Sobre el Cine Como Recurso Didáctico en Educación Infantil y Primaria. Ph.D. Thesis.

[31] Hernández-Nieto R.A. (2002). Contributions to Statistical Analysis.

[32] Ríos J.M., Matas A., Gómez E.R. (2014). Estudio sobre frecuencia del consumo de cine en estudiantes universitarios hispanoamericanos. Píxel-Bit.

[33] Campo L.R., Martínez V.A., Juanatey-Boga Ó. (2005). Evolución en las preferencias de los consumidores de productos audiovisuales. Comunicar.

[34] López N., Gómez L. (2012). Géneros, formatos y programas de televisión preferidos por los jóvenes. Análisis comparativo por comunidades autónomas. Icono 14.

[35] Marta-Lazo C.M., Gabelas-Barroso J.A.G. (2013). Hábitos de consumo televisivo de ficción entre los universitarios que estudian comunicación. Rev. Comun. SEECI.

[36] Sellés M. (2005). El documental: Un compromís polític. Trípodos.

[37] Pérez-Tornero J.M. (2008). La sociedad multipantallas: Retos para la alfabetización mediática. Comunicar.

[38] Fernández-Montalvo J., Peñalva A., Irazabal I. (2015). Hábitos de uso y conductas de riesgo en Internet en la preadolescencia. Comunicar.

[39] Ramos D., Ortega-Mohedano F. (2017). La revolución en los hábitos de uso y consumo de vídeo en teléfonos inteligentes entre usuarios Millenials, la encrucijada revelada. Rev. Lat. Comun. Soc..

[40] Masanet M.J., Scolari C.A. Analfabetisme Transmèdia: Aprofitant les Competències Transmèdia dels Adolescents a L’aula. https://www.raco.cat/index.php/RevistaPedagogia/article/view/100829.

[41] Aranda D., Roca M., Sánchez-Navarro J. (2013). Televisión e internet. El significado de uso de la red en el consumo audiovisual de los adolescentes. Quad. CAC.

[42] López N., Medina E., González P. (2013). Los jóvenes españoles demandan una televisión con más ficción y entretenimiento. Ámbitos.

[43] Gómez L., López N. (2014). La dieta mediática de los jóvenes españoles. Nuevos hábitos y tendencias de consumo en radio y televisión. En Caldevilla, D. (coord.). Parámetros actuales de evaluación para la comunicación persuasiva.

[44] García A., Tur-Viñes V., Pastor Y. (2018). Consumo mediático de adolescentes y jóvenes. Noticias, contenidos audiovisuales y medición de audiencias. Icono 14.

[45] AIMC (2017). Datos de Audiencia en España.

[46] López N., Gómez L. (2014). Nuevos hábitos de los jóvenes españoles y tendencias de futuro en el consumo de radio y televisión. Hist. Comun. Soc..

[47] Hernández J.F., Martínez M.A. (2016). Nuevos modelos de consumo audiovisual: Los efectos del binge-watching sobre los jóvenes universitarios. adComunica.

[48] Breu R. (2010). El Documental como Estrategia Educativa. De Flaherty a Michael Moore, diez Propuestas de Actividades.

[49] Guichot-Reina V., Merino M.A. (2016). Los cortometrajes de animación como herramienta didáctica para trabajar la educación en valores en educación infantil. Cuest. Pedagógicas.

